# Serum high-sensitivity C-reactive protein, amyloid-associated protein and N-terminal proBNP levels do not predict reversible myocardial ischaemia

**DOI:** 10.5830/CVJA-2010-041

**Published:** 2011-04

**Authors:** M Başkurt, F Aktürk, K Keskin, P Canbolat, A Yildiz, U Coskun, K Kilickesmez, O Esen, SK Muniboglu, B Karadag, A Kaya

**Affiliations:** Cardiology Department, Institute of Cardiology, Istanbul University, Haseki, Istanbul; Cardiology Department, Institute of Cardiology, Istanbul University, Haseki, Istanbul; Cardiology Department, Institute of Cardiology, Istanbul University, Haseki, Istanbul; Cardiology Department, Institute of Cardiology, Istanbul University, Haseki, Istanbul; Cardiology Department, Institute of Cardiology, Istanbul University, Haseki, Istanbul; Cardiology Department, Institute of Cardiology, Istanbul University, Haseki, Istanbul; Cardiology Department, Institute of Cardiology, Istanbul University, Haseki, Istanbul; Cardiology Department, Institute of Cardiology, Istanbul University, Haseki, Istanbul; Cardiology Department, Institute of Cardiology, Istanbul University, Haseki, Istanbul; Cardiology Department, Cerrahpasa Medical Faculty, Istanbul University, Haseki, Istanbul; Biochemistry Department, Institute of Cardiology, Istanbul University, Haseki, Istanbul

**Keywords:** brain natriuretic peptide, coronary artery disease, exercise, ischaemia

## Abstract

**Aim:**

The aim of this study was to detect any relationship between serum high-sensitivity C-reactive protein (hs-CRP), serum amyloid-associated protein (SAA) and N-terminal pro B-type natriuretic peptide (NT-proBNP) levels, and reversible myocardial ischaemia during cardiovascular exercise tests and to determine whether these biomarkers could predict transient myocardial ischaemia.

**Methods:**

Ninety-six patients (36 women, 60 men, mean age 57 ± 8.5 years) were included in the study. Venous blood samples were taken from patients before and 15 minutes after exercise testing. SAA and hs-CRP were analysed using immunonephelometric assays (Dade-Behring, BN II, Marburg, Germany). NT-proBNP (pg/ml) was determined using the immulite 1 000 chemiluminescence immunoassay system (Siemens Medical Solution Diagnostics, Deerfiled, USA). Forty-eight patients (18 women, 30 men) with positive exercise tests were allocated to the exercise-positive group and 48 (18 women, 30 men) with negative exercise tests were put in the exercise-negative group. Coronary angiography was performed on all patients in the exercise-positive group.

**Results:**

There was no difference between the levels of hs-CRP, SAA and NT-pro-BNP before and after exercise testing in both of the exercise groups.

**Conclusion:**

Serum levels of hs-CRP, SAA and NT-proBNP could not predict the occurrence of reversible myocardial ischaemia during exercise. Large-scale clinical studies are needed to clarify the status of hs-CRP, SAA and NT-proBNP with exercise.

## Summary

As a response to inflammatory cytokines, high-sensitivity C-reactive protein (hs-CRP) is synthesised mainly by the liver and to a lesser extent by macrophages and blood vessel walls. hs-CRP circulates freely in the plasma because there are no specific transporters for it.^1^,^2^ It has been shown that hs-CRP is a powerful predictor of cardiovascular adverse events and mortality in unstable coronary heart disease.^3^-^5^ However, its role in predicting transient myocardial ischaemia in stable coronary heart disease is not clear. While some studies found a strong relationship between serum hs-CRP levels and reversible myocardial ischaemia in exercise electrocardiographic testing,^6^ others did not find such a relationship.^7^,^8^

Serum amyloid-associated protein (SAA) is an acute-phase reactant and its concentration can reach up to 1 000-fold of the normal plasma concentration in the presence of inflammation. SAA is mainly synthesised by the liver.^9^ Serum levels of SAA rise with obesity, diabetes and the metabolic syndrome.^10^,^11^ Some studies point to a positive correlation between SAA and angiographically proven coronary artery disease, and serum concentration of SAA is reported to be a powerful predictor of cardiovascular events.^12^,^13^ It has been shown that patients with a high SAA concentration have more active atherosclerotic disease,^14^ but the relationship between serum SAA levels and transient myocardial ischaemia during exercise is not known.

B-type natriuretic peptide (BNP) is synthesised by the ventricular myocardium as a response to wall stress and is not stored.^15^ Myocardial ischaemia activates the pre-proBNP gene expression and secretion. Especially newly synthesised proBNP is secreted from the myocardium in response to ischaemia.^16^ BNP is the biologically active form and N-terminal proBNP (NT-proBNP) is the inactive form of proBNP. In one study, increase in serum BNP levels during electrocardiographic exercise testing was found in patients with stable angina pectoris. Also, the rise in serum BNP levels was correlated with the size of the ischaemic myocardium, assessed with positron emission tomography.^17^ In patients with angiographically proven coronary artery disease, serum NT-proBNP levels were related to the extent and severity of coronary artery disease.^18^

The purpose of this study was to detect any relationship between serum hs-CRP, SAA and NT-proBNP levels, and reversible myocardial ischaemia during cardiovascular exercise testing, and to determine whether these biomarkers could predict transient myocardial ischaemia.

## Methods

Ninety-six patients (36 women, 60 men, mean age 57 ± 8.5 years) were included in the study between March and May 2009. The study group comprised patients who had visited our outpatient clinic and electrocardiographic exercise testing was planned to detect ischaemia. All patients had a history of either exercise-induced angina or atypical angina. Patients with previous myocardial infarction, previous revascularisation, chronic kidney failure, known malignancy, serious peripheral arterial disease, heart failure, valvular heart disease, previous cerebrovascular accident, known chronic inflammatory conditions and with active infectious diseases were excluded from the study. The local ethics committee approved the study and informed written consent was obtained from all patients.

## Exercise testing

According to the current guidelines, patients discontinued medicines that could affect the exercise testing 48 hours before the test.^18^ Patients performed the exercise test after a three-hour fast and they were not allowed to drink tea/coffee or smoke before the test. All patients underwent a standard exercise stress test using the modified Bruce protocol.

Blood pressure, heart rate and 12-lead ECGs were recorded at rest, during each stage of exercise, at peak exercise, and for at least five minutes in the recovery phase. The ECG and ST-segment depression were continuously displayed and measured automatically by a computer-assisted system (Marquette-Case treadmill system, General Electric, Milwaukee, USA) in all 12 leads.

The subjects were exercised until one of the end-points was reached: age-specific target heart rate, or the development of symptoms necessitating termination of the test. Patients were encouraged to perform to their maximum effort and symptoms that developed during the test were recorded. The development of 0.10 mV (1 mm) or more of J-point depression measured from the PQ junction, with a relatively flat ST-segment slope (e.g. less than 0.7–1 mV/sec), depressed 0.10 mV or more 80 ms after the J point (ST 80) in three consecutive beats, with a stable baseline, was considered to be an abnormal response.

Criteria for a positive exercise test were determined as an abnormal ST-segment response, or other criteria for positive exercise testing [drop in systolic blood pressure > 10 mmHg from baseline blood pressure despite an increase in workload, when accompanied by other evidence of ischaemia; moderate to severe angina; sustained ventricular tachycardia; ST elevation (≥ 1.0 mm) in leads without diagnostic Q waves (other than V1 or avR)].^18^ Patients with a positive exercise test were placed in the positive exercise group. Patients who did not meet the positive criteria were not considered to have ischaemia and were put in the negative exercise group.

In order to detect the ischaemic aetiology, coronary angiography was performed on an outpatient basis on all patients in the positive exercise group on another suitable day. Stenosis that reduced the lumen diameter by 50% in a coronary artery was accepted as a haemodynamically significant lesion.

## Blood sampling and biochemical analysis

Venous blood samples were taken from the antecubital veins of patients before and 15 minutes after the exercise test. Blood samples were taken in standard heparinised and non-heparinised tubes. Samples were centrifuged at 3 500 rpm for 10 minutes. Care was taken with the blood samples so as not to become lipaemic or haemolysed. Centrifuged heparinised blood samples were kept at –20°C.

SAA and hs-CRP were immediately assayed from the non-heparinised blood samples using immunonephelometric assays (Dade-Behring, BN II, Marburg, Germany). The reference concentration for SAA was < 5 mg/l and for hs-CRP it was < 3mg/l. After collecting the samples, NT-proBNP (pg/ml) was assayed using the immulite 1 000 chemiluminescence immunoassay system (Siemens Medical Solution Diagnostics, Deerfiled, USA).

## Statistical analysis

SPSS for Windows version 13.0 was used for statistical analysis. Student’s t-test was used for comparison of mean values; *p* < 0.05 was accepted as significant.

## Results

Of the 96 patients, 48 (18 women, 30 men) with positive exercise tests were allocated to the positive exercise group and 48 (18 women, 30 men) with negative exercise tests were put into the negative exercise group. There was a statistically significant difference between the two groups for smoking, hyperlipidaemia, hypertension and family history of coronary heart disease (Table 1).

**Table 1.** Demographic Features And Risk Factors Of Patients

	*Positive exercise testing (n = 48) (%)*	*Negative exercise testing (n = 48) (%)*	p*-value*
Age (years)	57 ± 10.0	57 ± 7.0	> 0.05
Gender (female/male)	18/30	18/30	> 0.05
BMI (kg/m^2^)	27.7 ± 3.7	28.9 ± 4.2	> 0.05
Diabetes mellitus	16/48 (33)	14/48 (29)	> 0.05
Current smoker	30/48 (62)	18/48 (37)	< 0.03
Hypertension	44/48 (91)	18/48 (37)	< 0.001
Hyperlipidaemia	44/48 (91)	26/48 (54)	< 0.001
Family history	41/48 (85)	16/48 (33)	< 0.001

BMI: body mass index.

Mean exercise duration was significantly longer in the negative exercise group than in the positive exercise group (11.5 ± 1.1 vs 8.9 ± 2.6 min, *p* < 0.001). Metabolic equivalents (METs) were also higher in the negative exercise group than in the positive group (12.3 ± 1.5 vs 9.5 ± 2.6, *p* < 0.001). There was no difference between the levels of hs-CRP, SAA and NT-proBNP before and after exercise testing in both groups (Table 2).

**Table 2.** Serum HS-CRP, SAA And NT-proBNP Levels Before And After Exercise Testing

	*Positive exercise testing (n = 48)*		*Negative exercise testing (n = 48)*		
	*Pre-test*	*Post-test*	*Pre-test*	*Post-test*	p*-value*
Hs-CRP (mg/l)	4.1 ± 6.2	4.9 ± 6.5	1.9 ± 1.3	2.7 ± 1.7	> 0.05
SAA (mg/l)	8.4 ± 12.3	10.0 ± 14.0	5.8 ± 4.2	6.2 ± 4.1	> 0.05
NT-proBNP (pg/ml)	175.1 ± 392.3	201.5 ± 461.6	92.2 ± 130.5	102.5 ± 139.2	> 0.05

hs-CRP: high-sensitivity C-reactive protein, SAA: serum amyloid-associated protein, NT-proBNP: N-terminal proBNP.

The results of coronary angiography in the positive exercise group of patients were as follows: there was single-vessel disease in 21 patients, two-vessel disease in 11 patients, isolated side-branch disease in five patients, triple-vessel disease in four patients, left main coronary artery disease in two patients, left main plus triple-vessel disease in two patients, coronary artery ectasia in two patients, and spontaneous dissection of one coronary artery in one patient.

Medical follow up was chosen in 28 patients as the main therapeutic modality, whereas 14 patients underwent percutaneous coronary intervention and were discharged one day after the procedure with appropriate medication. In six patients, an aortocoronary bypass grafting operation was successfully performed. No surgery-related complications occurred and all of the patients were discharged uneventfully.

## Discussion

In this study, we observed a slight non-significant rise in the serum levels of hs-CRP, SAA and NT-proBNP with exercise. Although the increase in serum SAA, hs-CRP and NT-proBNP levels during exercise testing was slightly more in the positive exercise group, the difference was not significant. We concluded that serum levels of hs-CRP, SAA and NT-proBNP could not predict the occurrence of reversible myocardial ischaemia during exercise.

Shehadeh *et al.* reported that there was no relation between hs-CRP and transient myocardial ischaemia in patients with a history of chronic heart failure or previous myocardial infarction.^7^ They also found that exercise duration was longer in patients with lower levels of hs-CRP. In our study, although basal serum hs-CRP levels were the same in the two groups, the exercise capacity of the non-ischaemic group was better than in the ischaemic group. Veleska *et al.* could not find any relationship between serum hs-CRP levels and exercise testing results in a study that included 200 patients with positive exercise tests.^8^ On the other hand, Cosin-Sales *et al.* reported a correlation between serum hs-CRP levels and ST-segment depression during exercise testing with Holter monitoring, and the frequency of angina episodes in patients with typical chest pain and normal coronary angiograms.^6^ Therefore, the relationship between hs-CRP and reversible myocardial ischaemia was not as clear as in unstable coronary artery disease and needs to be clarified in large-scale studies.

High-density lipoprotein (HDL) is the main carrier of SAA in plasma. In cases of low HDL status, SAA is carried by the other apolipoproteins.^19^-^21^ SAA may comprise up to 80% of HDL during inflammation, which results in deterioration of the anti-atherogenic properties of HDL.^19^-^21^ Ogasawara *et al*. reported that HDL molecules rich in SAA are a risk factor for cardiovascular diseases, therefore the SAA/LDL complex was found to be directly related to high cardiovascular risk.^22^

In both groups in our study, serum SAA levels increased slightly after exercise testing but there was no significant difference. The increase in SAA levels seemed not to be associated with myocardial ischaemia. We also could not find any study addressing the relationship between SAA and transient myocardial ischaemia in the literature.

Early studies reported a decrease in serum inflammatory biomarker levels in subjects who exercised regularly.^23^ However, during short, sudden exercise, the immune system becomes activated and inflammatory biomarker levels elevate in the serum. This mechanism has not been clarified.^23^ The possible mechanisms are: muscle damage during exercise, new developing oxidative stress as a response to the increase in oxygen demand with the forced usage of muscles, and increased interleukine-6 (IL-6) synthesis in muscles.

Firstly, the increase in serum CRP and SAA levels during exercise may be due to increased synthesis of IL-6. In addition, leucocyte counts increase during exercise.^24^ As a result, these changes may trigger immune system activation and increased levels of acute-phase reactants. In our study, serum SAA levels increased, as did serum hs-CRP levels after exercise in both the ischaemic and non-ischaemic groups. But we felt these elevations were as a result of exercise-induced immune system activation and did not reflect myocardial ischaemia.

NT-proBNP is the inactive form of BNP. Like other natriuretic peptides, the serum concentration of NT-proBNP increases as a response to increased left ventricular myocardial wall stress.^25^,^26^ NT-proBNP levels increased in patients with disorders causing left ventricular diastolic dysfunction, but this increase in NT-proBNP was not as much as that seen in left ventricular systolic dysfunction.^27^

In recent years, increased NT-proBNP synthesis has been shown as a response to myocardial ischaemia. In addition, it has also been shown that increases in NT-proBNP levels were related to the extent and severity of the coronary artery disease.^28^ Serum NT-proBNP was found to be a strong predictor for future cardiovascular events in patients with stable angina pectoris.^29^

Some studies were designed for detecting the correlation between NT-proBNP and transient myocardial ischaemia. Kurz *et al.* found that baseline NT-proBNP levels were higher in patients with transient ischaemia; however there was no correlation between NT-proBNP levels and ischaemia after exercise testing.^30^ In another study which was done with myocardial perfusion scintigraphy, Staub *et al.* reported that NT-proBNP levels before and after exercise were significantly higher in patients who developed transient ischaemia.^31^ Sabatin *et al.* and Foote *et al*. also reported similar results.^32^,^33^ On the other hand, other studies reported contradictory findings.^34^,^35^ In a study, before and after exercise testing, serum NT-proBNP levels were studied hourly for six hours and NT-proBNP levels were not found to be correlated with myocardial ischaemia.^36^

In our study, we could not find any relationship between transient myocardial ischaemia and serum NT-proBNP levels before and after exercise testing. In both ischaemic and non-ischaemic patients, serum NT-proBNP levels increased slightly after exercise testing. This might have been caused by a mechanism other than ischaemia. In a study on healthy adults, an increase in serum NT-proBNP levels was shown after electrocardiographic exercise testing.^37^ It was concluded that these increases after exercise testing were due to elevated cardiac load and increased ventricular wall tension.^38^,^39^

The most important limitation of our study was the small number of patients. This was due to us excluding patients with known coronary artery disease and prior revascularisation. Another limitation was that it was a single-centred study reflecting a local area.

## Conclusion

In our study we found that there was no relationship between exercise-induced transient myocardial ischaemia and serum hs-CRP and SAA levels as inflammatory biomarkers, and serum NT-proBNP levels as a non-inflammatory biomarker. All three biomarkers increased with exercise for different reasons but these elevations were not related to transient myocardial ischaemia. Large-scale clinical studies are needed to clarify the status of hs-CRP, SAA and NT-proBNP with exercise.
